# *COVID-19 Stats:* COVID-19 Incidence,* by Age Group^†^ — United States, March 1–November 14, 2020^§^

**DOI:** 10.15585/mmwr.mm695152a8

**Published:** 2021-01-01

**Figure:**
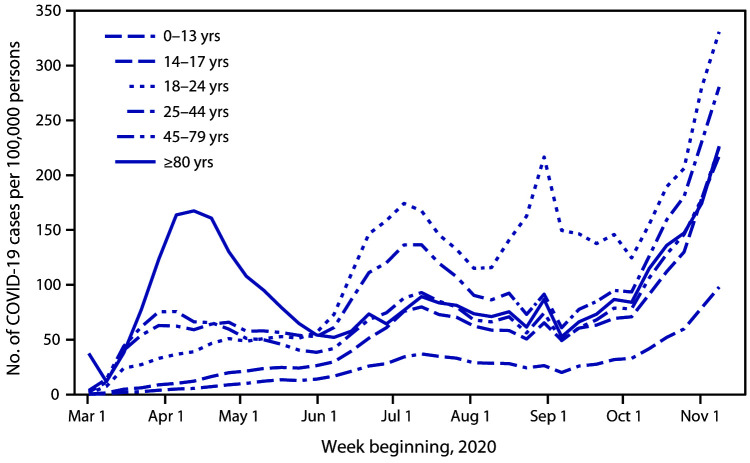
During late March–late May, COVID-19 incidence was highest among adults aged ≥80 years, with a peak in incidence in the week beginning April 12. In June, incidence increased in all age groups, with the most rapid rate of increase and highest overall incidence among young adults aged 18–24 years; the rate in this group continues to be the highest among all age groups. Incidence steadily increased among children and adolescents (aged 0–17 years). The incidence in high school–aged persons (aged 14–17 years) was markedly higher than that in younger children by early July, then decreased before increasing in September. During late September–early October, weekly incidence decreased among young adults aged 18–24 years only, then continued to steadily increase among all age groups through November 14.

**Source:** CDC COVID-19 case-level report forms, March 1–November 14, 2020.

